# Evolution of Material Properties and Residual Stress with Increasing Number of Passes in Aluminium Structure Printed via Additive Friction Stir Deposition

**DOI:** 10.3390/ma17143457

**Published:** 2024-07-12

**Authors:** Vladislav Yakubov, Halsey Ostergaard, James Hughes, Evren Yasa, Michail Karpenko, Gwénaëlle Proust, Anna M. Paradowska

**Affiliations:** 1 School of Civil Engineering, The University of Sydney, Sydney, NSW 2006, Australia; halsey.ostergaard@sydney.edu.au (H.O.); gwenaelle.proust@sydney.edu.au (G.P.); 2 Australian Nuclear Science and Technology Organisation, Kirrawee, NSW 2234, Australia; 3 Advanced Manufacturing Research Centre North West (AMRC NW), University of Sheffield, Blackburn BB2 7HP, UK; j.hughes@sheffield.ac.uk (J.H.); e.yasa@sheffield.ac.uk (E.Y.); 4 Heavy Engineering Research Association, Manukau City Centre, Auckland 2104, New Zealand; mkarpenko@hera.org.nz; 5 Sydney Manufacturing Hub, The University of Sydney, Sydney, NSW 2006, Australia; 6 School of Aerospace, Mechanical and Mechatronic Engineering, The University of Sydney, Sydney, NSW 2006, Australia

**Keywords:** additive manufacturing, additive friction stir deposition, aluminium alloy, residual stress, hardness, microstructure

## Abstract

Additive friction stir deposition (AFSD) is an emerging solid-state additive manufacturing process with a high deposition rate. Being a non-fusion additive manufacturing (AM) process, it significantly eliminates problems related to melting such as cracking or high residual stresses. Therefore, it is possible to process reactive materials or high-strength alloys with high susceptibility to cracking. Although the residual stresses are lower in this process than with the other AM processes, depending on the deposition path, geometry, and boundary conditions, residual stresses may lead to undesired deformations and deteriorate the dimensional accuracy. Thermal cycling during layer deposition, which also depends on the geometry of the manufactured component, is expected to affect mechanical properties. To this day, the influence of the deposit geometry on the residual stresses and mechanical properties is not well understood, which presents a barrier for industry uptake of this process for large-scale part manufacturing. In this study, a stepped structure with 4, 7, and 10 passes manufactured via AFSD is used to investigate changes in microstructure, residual stress, and mechanical property as a function of the number of passes. The microstructure and defects are assessed using scanning electron microscopy and electron backscatter diffraction. Hardness maps for each step are created. The residual stress distributions at the centreline of each step are acquired via non-destructive neutron diffraction. The valuable insights presented here are essential for the successful utilisation of AFSD in industrial applications.

## 1. Introduction

Additive friction stir deposition (AFSD) offers unique advantages over other additive manufacturing (AM) processes. It is capable of fabricating large structures due to its high deposition rate [1,2]. Furthermore, the only consumable is the feedstock material, and no inert gas is necessary for processing aluminium, thus presenting high cost effectiveness and reduced environmental impact of equipment operation [3,4]. The layer-by-layer solid-state AFSD process allows for flexibility in structure design and the use of wrought aluminium alloy feedstock [5], which, in fusion processes, experiences solidification cracking and deformation due to thermal-gradient-induced residual stresses and Scheil solidification [6,7].

Research on AFSD is mainly focused on Al alloys, although it has also been successfully applied to a broad range of materials such as Ti alloys [8,9], Mg alloys [10], Cu [11], high-entropy alloys (HEAs) [12,13], stainless steel [14,15], and high-tensile steel [16]. By avoiding the liquification and solidification of the material, AFSD avoids element loss [17], porosity [18], and cracking [7], which are known to occur in fusion AM processes. Furthermore, extreme plastic deformation during AFSD results in a deposit with a refined equiaxed grain structure, and research on the direct recycling of aluminium alloy waste from subtractive manufacturing processes has demonstrated that extreme plastic deformation breaks up intermetallic particles and oxides [19,20,21].

Despite these advantages, additional research is needed to understand microstructure control and the residual stress profile in the as-manufactured structure, which would improve the acceptance of AFSD by industry. In the case of precipitation-strengthened aluminium alloy grades, AFSD leads to a hardness gradient, which is caused by the thermal history affecting the precipitation strengthening mechanism and appears to be influenced by the structure height [22,23,24]. Garcia et al. [25] have shown that the processing temperature during AFSD approaches the solidus temperature of the Al-Mg-Si alloy, while Phillips et al. [26] have demonstrated that the high temperature under the rotating tool during deposition causes dissolution of the β″-phase, resulting in the loss of the precipitation strengthening mechanism. Meanwhile, Tang et al. [23] reported that Q′ and β′ precipitates form in the deposit from the solid solution due to the persistent heat input of the multilayer deposition process; these do not provide effective strengthening. Since precipitate evolution is a thermal- and time-driven process [27,28], the AFSD deposit height is expected to play a key role in the hardness gradient, since the first deposited layers will be exposed to an elevated temperature for an increased time as the deposit height increases. However, the effect of the deposit height on the hardness gradient of an as-manufactured AFSD Al6061 structure has not been thoroughly explored, with existing research mainly reporting single-line hardness scans.

With the high deposition rate and open processing environment of AFSD [2], it has become possible to additively manufacture large-scale components that would traditionally be manufactured via subtractive processes, achieving significant savings via reduced material waste. Furthermore, waste chips from subtractive manufacturing processes can be used to make feedstock for AFSD, presenting an additional avenue for cost reduction while also increasing manufacturing sustainability [4,19,20,21]. AFSD has already been applied to manufacture large aluminium structures such as a 6.1 × 9.1 × 3.7 m^3^ hull [29] and a 3.05 m diameter cylinder [30]. The design freedom of AFSD could allow increased flexibility in building design by realising complex and aesthetically pleasing load-bearing joints [31] or even composite/dissimilar joints [32]. However, the welding of large structures is generally problematic due to residual stresses causing structure deformation [33], and external loads superimpose over residual stresses, thus affecting the load-bearing capacity [34]. In the case of AFSD, the effects of different variables on the residual stresses formed during manufacturing have not been studied in detail.

Neutron diffraction residual stress measurements conducted by Zhu et al. [35] on a 66 mm high AFSD Al6061 deposit manufactured using an omnidirectional deposition path showed compressive residual stresses in the finishing end of the deposit and tensile residual stresses in the starting and centre regions. The highest residual stress was approximately 150 MPa (tensile) in the longitudinal direction and was located at 10 mm in the substrate from the deposit/substrate interface. Meanwhile, for a 10.5 mm high Al6061 deposit, Yakubov et al. [36] noted negligible and slightly compressive residual stresses in the deposit, and the highest residual stress (tensile) measured was 74 MPa in the longitudinal direction at 2.5 mm in the substrate from the deposit/substrate interface. Based on these data, it appears that the residual stress distribution is affected by the deposit height.

This research investigates the microstructure, hardness, defects, and residual stress distribution in a stepped AFSD as-manufactured Al6061 structure with an increasing number of passes. These insights contribute to ongoing efforts to provide information on AFSD structure characteristics to drive the uptake of this innovative manufacturing process in the large-scale manufacturing industry.

## 2. Experimental Section

**Figure 1:**
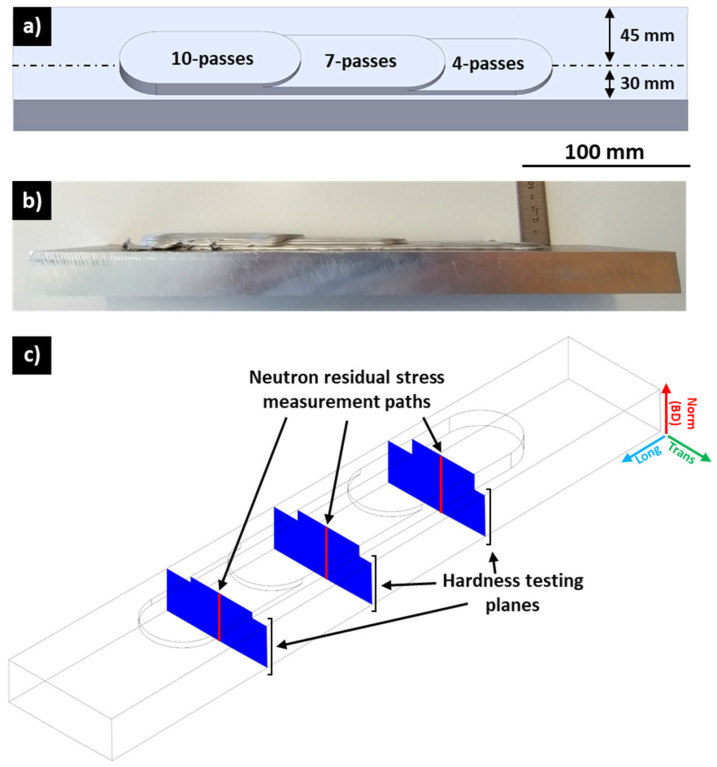
(**a**) Three-dimensional render of investigated AFSD-manufactured structure showing top ¾ view; (**b**) side view of as-manufactured sample; (**c**) illustration of hardness testing planes (blue) and neutron residual stress measurement paths (red).

AFSD was conducted using a MELD L3 machine (Christiansburg, VA, USA) at The University of Sheffield’s, Advanced Manufacturing Research Centre North West (AMRC NW, Blackburn, UK). As seen in Figure 1a,b, three deposits with heights 3.5 mm, 6.5 mm, and 9.5 mm corresponding to 4 passes, 7 passes, and 10 passes, respectively, were manufactured on the same Al6061-T6 substrate with the dimensions 26 × 78 × 400 mm^3^ using a 38 mm diameter AFSD tool with a flat contact surface and bidirectional deposition. The substrate was clamped at each of the four corners. The feedstock rod was composed of Al6061-T6 with a square cross-section of 9.5 × 9.5 mm^2^. The layer height for the first deposit onto the substrate was 0.5 mm, and, for subsequent layers, it was 1 mm. The resulting deposit width at the interface was 42.2 mm. The tool rotation was set at 300 RPM; the material feed rate was 152 mm·min^−1^; the tool in-plane movement speed was 381 mm·min^−1^; and the tool out-of-plane movement speed was 8.9 mm·min^−1^.

Prior to optical microscopy, SEM (scanning electron microscopy), SEM-EBSD (scanning electron microscopy-electron backscatter diffraction), and SEM-EDS (scanning electron microscopy-energy dispersive X-ray spectroscopy), the samples underwent mechanical grinding down to 4000-grit SiC, followed by polishing with a 0.05 µm colloidal silica suspension. Samples for SEM, SEM-EBSD, and SEM-EDS underwent additional vibratory polishing for two hours in a 0.05 µm colloidal silica suspension. Optical microscopy utilised the Hirox MXB-050Z (Tokyo, Japan), while hardness maps were obtained on the same surfaces as shown in Figure 1c via a Struers DuraScan-80 automated Vickers hardness tester (Ballerup, Copenhagen, Denmark) in accordance with ASTM E92-23 [37] with 500 g force for 10 s per indent and approximately 1 mm indent separation (greater than 4× the distance of the indent diagonal). ImageJ analysis software (version 1.54j) was used to determine the void area, total deposit area, and inclusion diameter from 2D optical microscopy images.

The neutron diffraction (ND) residual stress measurement was conducted using a Kowari neutron strain scanning instrument at the Open Pool Australian Light-water (OPAL) reactor at the Australian Nuclear Science and Technology Organisation (ANSTO). The instrument was setup to utilise a 1.7 Å monochromatic neutron wavelength to capture the Al(311) reflection for longitudinal, transverse, and normal residual stress measurements at the centreline of each deposit step height (Figure 1c). The gauge volume for the neutron beam was set at 1 × 1 × 1 mm^3^. The triaxial residual stress measurement line scans were positioned along the centreline of each deposit step in the steady state of the process, spanning from the final deposit layer to the bottom of the substrate. A stress-free reference specimen cut from an AFSD structure manufactured under similar conditions was measured at the same time as the 4-pass, 7-pass, and 10-pass samples to determine the stress-free d_0_ value.

## 3. Results and Discussion

### 3.1. Defects and Microstructure

**Figure 2:**
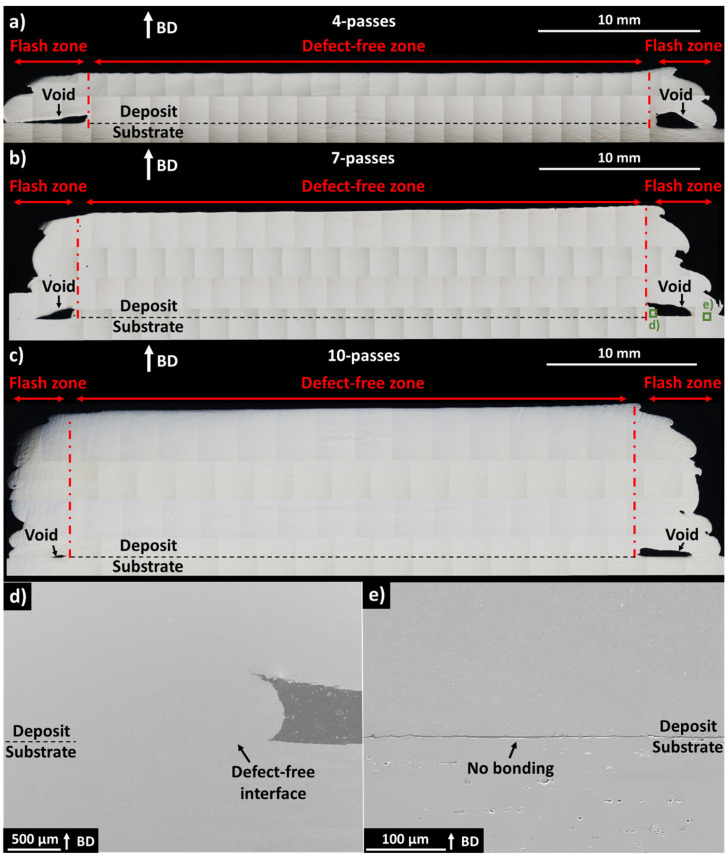
Optical images showing cross-section of (**a**) 4-pass deposit, (**b**) 7-pass deposit, and (**c**) 10-pass deposit. SEM images showing (**d**) defect-free deposit and substrate interface and (**e**) defective deposit and substrate interface, with locations provided in (**b**). BD indicates build direction.

As seen in Figure 2a–c, all deposit heights exhibited a defect-free central zone and a flash zone at the sides. In each case, the defect-free central zone comprised the majority of the deposit and spans across the deposit centreline. Here, bonding to the substrate appeared excellent, and no cracking, porosity, or other defects were noted. Meanwhile, a flash zone was apparent at the edges of each deposit, which appeared to be caused by swirling of the extruded material beyond the rotating tool edges. In the flash zone, voids were visible as well as the absence of good bonding to the substrate. It was apparent that the voids at the flash zone became smaller with increasing deposit height. For the 4-pass deposit (Figure 2a), 7-pass deposit (Figure 2b), and 10-pass deposit (Figure 2c), the voids presented between the deposit and substrate at the left edge had an area of 1.03 mm^2^, 0.87 mm^2^, and 0.11 mm^2^, respectively. Table 1 shows the calculated 2D surface area of the defect-free, left-edge flash, and right-edge flash zones of the 4-pass, 7-pass, and 10-pass deposits in Figure 2a–c. While the left-edge and right-edge flash areas increased with deposit height, as a percentage of the total area, they did not follow an obvious trend with increasing deposit height. They remained similar for all deposit heights, suggesting that the factors influencing flash formation are not strongly dependent on the deposit height. To achieve a complete defect-free structure, it is necessary to remove the left- and right-edge flash zone via machining, resulting in the retention of 82–86.3% (Table 1) of the original deposit material as calculated from the 2D area.

SEM images of the seven-pass deposit and substrate interface at the edge of the defect-free zone (Figure 2d) confirmed that the interface between the deposit and substrate did not contain separation or cracking and appeared defect free, even at the far end of the defect-free zone. However, at the flash zone (Figure 2e), the interface between the deposit and substrate was characterised by a microscale separation not visible in optical microscopy images, alluding to no or poor load-carrying ability. While bonding defects such as the kissing bond and the hooking defect have been characterised for friction stir welding (FSW), bonding defects and their mechanisms have not yet been fully characterised for AFSD [36].

Garcia et al. [25] analysed the material flow of the Al6061 alloy during AFSD using a rotating tool with a flat contact surface and noted that the feedstock material must be extruded and then fill the area under the rotating tool during the deposition of the first layer. Furthermore, the material is in a compression-dominated state under the feedstock extrusion hole, applying significant force into the substrate. However, as the material is extruded and flows away from the extrusion hole, it transitions to a shear-dominated state, resulting in decreased force into the substrate. Meanwhile, Zeng et al. [38] performed AFSD of Al6061 with a rotating tool contact face containing water-drop-shaped 2.3 mm height inner pins and 1.8 mm height outer pins. Similar to Garcia et al. [25], they noted a compression-dominated area in the substrate under the path that the feedstock extrusion hole traversed. However, the substrate area under the rotating tool but away from the feedstock extrusion hole exhibited a hook-barb-like tool-stirred profile, caused by interaction of the substrate with the pins during the first layer’s deposition. Nevertheless, at the flash region, which was not under the rotating tool, gaps between the deposit and substrate were also noted.

Based on these results, it is expected that near to and beyond the rotating tool edge, insufficient force into the substrate or previous layer is present for deposit mixing and the formation of a metallurgical bond. This is supported by the observed defect-free zone span of 34.7 mm, 35.0 mm, and 37.4 mm for the 4-pass, 7-pass, and 10-pass deposits, respectively, which is less than the 38 mm AFSD tool diameter. The AFSD tool diameter is, thus, seen as the upper limit for the defect-free zone span. It should be noted that current research has focused on 2D methods to identify defects, only providing information at the steady-state position of the deposit. The behaviour of the voids and defects in relation to the start and end of the deposit path is, therefore, not yet fully understood. In FSW, non-destructive computed tomography revealed that defects may form only at certain parts of the weld, e.g., at the weld start or at a distance from the weld start [39,40,41], due to the transient temperature [41,42].

**Figure 3:**
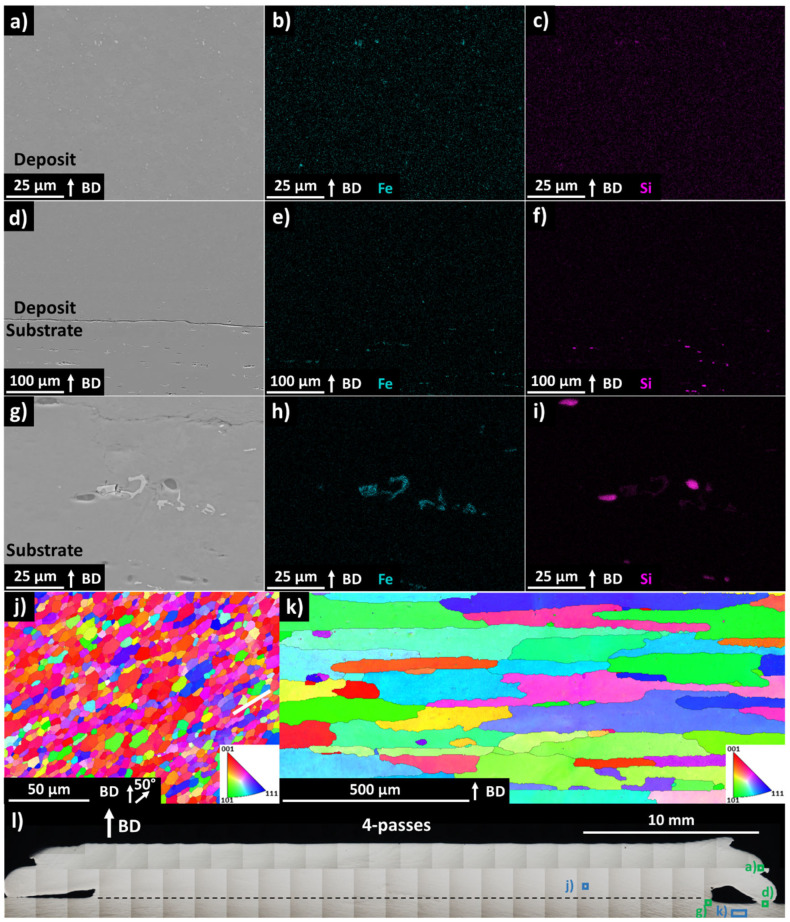
SEM image of deposit and EDS images showing Fe and Si distribution are provided as (**a**), (**b**), and (**c**), respectively, with imaged location provided in (**l**). SEM image of deposit and substrate interface and EDS images showing Fe and Si distribution are provided as (**d**), (**e**), and (**f**), respectively, with imaging location provided in (**l**). SEM image of substrate and EDS images showing Fe and Si distribution are provided as (**g**), (**h**), and (**i**), respectively, with imaging location provided in (**l**). (**j**) EBSD of deposit at location provided in (**a**); (**k**) EBSD of substrate at location provided in (**l**). BD indicates build direction.

SEM-EDS images of the four-pass deposit and substrate (Figure 3a–i) indicated inclusions containing Fe and Si, which were present in all the imaged locations, and the size and morphology of these inclusions were different in the deposit relative to the substrate. At the deposit (Figure 3a,b), the inclusions were circular and less than 3 µm in diameter, while at the substrate (Figure 3g,h) the Fe-containing inclusions exhibited an irregular morphology with 8 µm equivalent diameter, and the circular Si inclusions exhibited a 6 µm diameter. SEM-EDS of the deposit and substrate interface at the flash zone (Figure 3d–f) showed the inclusion size difference between the deposit and substrate with no apparent transition zone, confirming that no mixing between the deposit and substrate material occurred in this area.

Fe is a common impurity element in Al6061, and the formation of AlFeSi and AlFeMn phases has been reported in traditionally processed Al6061 [43,44], while Al(MnCrFe)Si has been confirmed in Al6061 AFSD deposits [23]. These intermetallic phases are known to be hard and brittle, with the load redistribution from large particles inducing bulk material embrittlement [43,45]. However, the extreme plastic deformation during AFSD causes the breakup and refinement of such intermetallics as well as oxide inclusions, resulting in increased ductility [4,21,23].

SEM-EBSD images of the four-pass deposit and substrate indicated the presence of a highly refined grain structure in the deposit (Figure 3j) with an 18 µm equivalent circular grain diameter (ECD) and minor elongation 50° away from the build direction. In the substrate (Figure 3k), the grains were seen to be large and columnar with a 194 µm ECD and elongation perpendicular to the deposit’s build direction. The grain geometry of the substrate is likely caused by unrelated rolling or extrusion during the substrate manufacturing process and is not a result of AFSD. For AFSD, the deposit height did not appear to influence the grain size within the test conditions applied in this study [35,46].

### 3.2. Hardness Distribution

**Figure 4:**
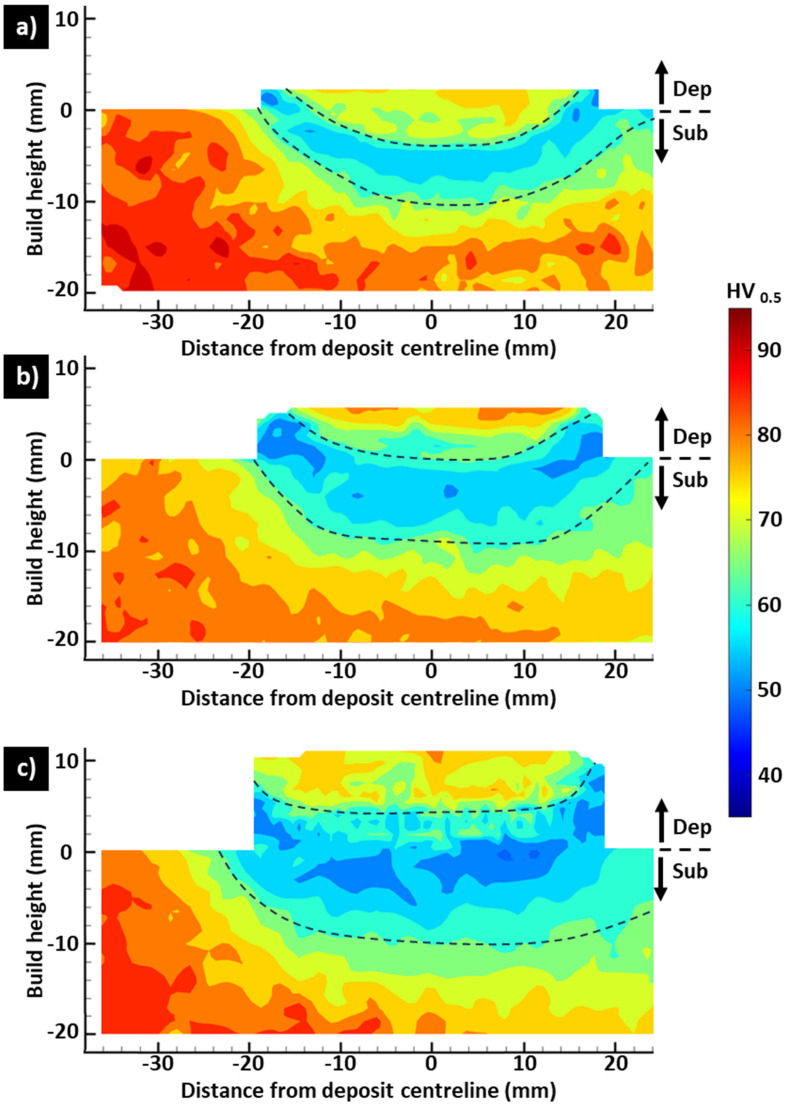
Hardness distribution for (**a**) 4-pass, (**b**) 7-pass, and (**c**) 10-pass deposits. The region between the dashed lines is the low-hardness zone.

The hardness distribution for the 4-pass (Figure 4a), 7-pass (Figure 4b), and 10-pass (Figure 4c) deposits indicated that material softening occurred in the AFSD process for all cases, with an increased deposit height resulting in a larger area of lower hardness. While the average hardness of the Al6061-T6 feedstock and substrate were measured to be 104 HV_0.5_, such high hardness was not noted in any region of the as-deposited structures. Furthermore, a trend was noted in which the final deposit layer (top of deposit) exhibited moderate hardness in the range of 65–83 HV_0.5_. This hardness zone was surrounded by a U-shaped area of low hardness in the range of 47–60 HV_0.5_, with a gradual transition between the moderate- and low-hardness zones.

For each deposit, the moderate-hardness zone spanned approximately 6 mm from the top at the centreline and was not significantly affected by the deposit height. Meanwhile, at the deposit centreline and in the build direction, the low-hardness zone was 7 mm wide for the 4-pass deposit (Figure 4a), 9 mm wide for the 7-pass deposit (Figure 4b), and 15 mm wide for the 10-pass deposit (Figure 4c), indicating an increase in the area of the low-hardness zone with the increase in deposit height.

Furthermore, the deposit placement was offset from the substrate centreline as shown in Figure 1a and reflected in Figure 4a–c. Increased softening was seen to occur on the right side of the substrate in Figure 4a–c relative to the left side of the substrate, corresponding with the deposit offset direction. Surprisingly, the refined grains in the AFSD deposit relative to the substrate did not obviously result in a hardness increase, indicating that the Hall–Petch strengthening mechanism cannot compensate for the loss of the precipitation strengthening mechanism during AFSD [47].

**Figure 5:**
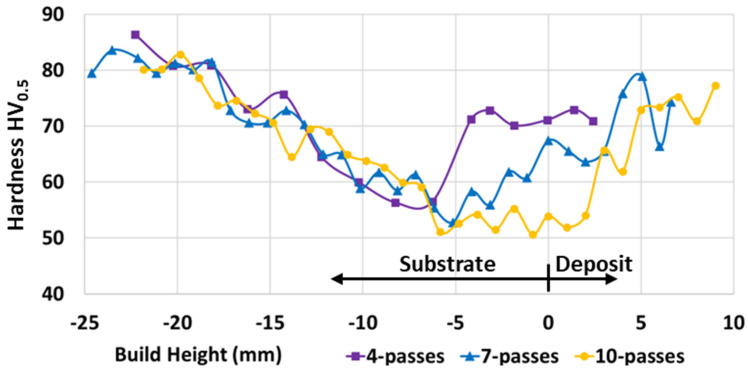
Hardness along deposit centreline for 4-pass, 7-pass, and 10-pass deposits.

Figure 5 shows the centreline hardness for the 4-pass, 7-pass, and 10-pass deposits. In this figure, the hardness for all deposit heights demonstrates similar behaviour from 6 mm into the substrate to the bottom of the substrate. However, from 6 mm into the substrate to the top of the deposit, each deposit height presents a different behaviour, with the 4-pass deposit demonstrating less softening at the same absolute height than the 7-pass deposit, and the 7-pass deposit demonstrating less softening at the same absolute height than the 10-pass deposit.

The cause of the hardness gradient is the thermal history during AFSD processing. Qiao et al. [48] used the thermocouple located in the AFSD rotating tool to investigate the effect of the AFSD tool’s rotation speed on the thermal history during eight-pass deposition of Al6061 feedstock on an Al6061 substrate. They noted an average tool temperature of 480.7 °C, 493.8 °C, and 500.9 °C for 450 RPM, 500 RPM, and 550 RPM tool rotation speeds, respectively. Meanwhile, Ghadimi et al. [49] placed a thermocouple in the Al6061 substrate prior to the deposition of Al6061 feedstock examining 1 mm, 2 mm, and 3 mm layer thickness for the manufacturing of ~40 mm height deposit. For a 1 mm layer thickness, the average substrate temperature increased with the deposit height, and, during the deposition of the final layers, it was approximately 245 °C. Meanwhile, the 2 mm and 3 mm layer thicknesses exhibited a lower average substrate temperature compared to that for the 1 mm layer thickness for all but the first deposited layers. For the 2 mm and 3 mm layer thicknesses, the average substrate temperature during the deposition of the final layers was approximately 160 °C and 145 °C, respectively. These results indicate that process parameters have a significant influence on the thermal history. 

Furthermore, such temperatures are sufficient for the dissolution of β″ strengthening precipitates and the growth of precipitates that do not provide effective strengthening. Using transmission electron microscopy with energy-dispersive X-ray spectroscopy (TEM-EDX), Tang et al. [23] noted that the final layer of an 18-pass (72 mm height) AFSD structure contained only the Al(MnCrFe)Si intermetallic phase, while the middle and bottom layers of the deposit contained Q′, Al(MnCrFe)Si, and β′.

From Figure 4a–c, it is noticed that the right side of the deposit contained a wider low-hardness zone due to the deposit offset as previously discussed in this section, indicating that the deposit and substrate geometries influence the thermal history, resulting precipitate formation, and hardness. A similar hardness reduction was previously noted for the FSW of precipitation-strengthened aluminium alloys, and water cooling effectively the reduced softening by lowering the peak processing temperature, thus inhibiting solute atom diffusion [50,51]. The application of thermal management to AFSD may also be beneficial to prevent the observed material softening. However, to the author’s knowledge, a detailed investigation of this topic has not yet been conducted. Instead, the elimination of such a hardness gradient in AFSD aluminium alloys has been reported by applying a post-process heat treatment [21,52,53].

### 3.3. Residual Stress Distribution

**Figure 6:**
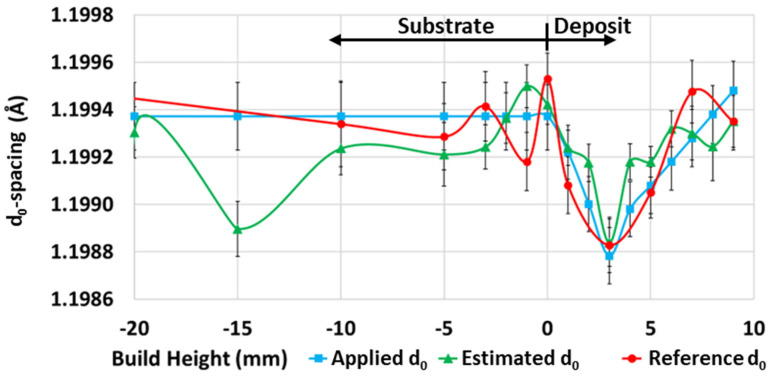
The d_0_∔spacings determined from ND data. Applied d_0_ refers to values used for residual stress calculation. Estimated d_0_ was calculated from 10-pass deposit data by assuming strain-free normal principal direction and was used for comparison with reference d_0_ value. Reference d_0_ is raw value determine from strain-free reference sample.

The position-dependent unstressed lattice spacing (d_0_-spacing) was calculated from an unstressed reference sample and compared to the estimated d_0_ determined by assuming the stress-free normal principal direction in the 10-pass deposit. In both cases, the smallest lattice spacing was noted at a 3 mm deposit height, and it increased approximately linearly towards the substrate interface and towards the top of the deposit. From the deposit interface to the bottom of the substrate, an approximate constant d_0_ value was noted in the reference specimen, which was expected since no deformation or other processing occurred in this area. As such, an applied d_0_ value was determined, which was used to calculate the residual stresses for 4-pass, 7-pass, and 10-pass deposits. The d_0_-spacings are presented in Figure 6.

**Figure 7:**
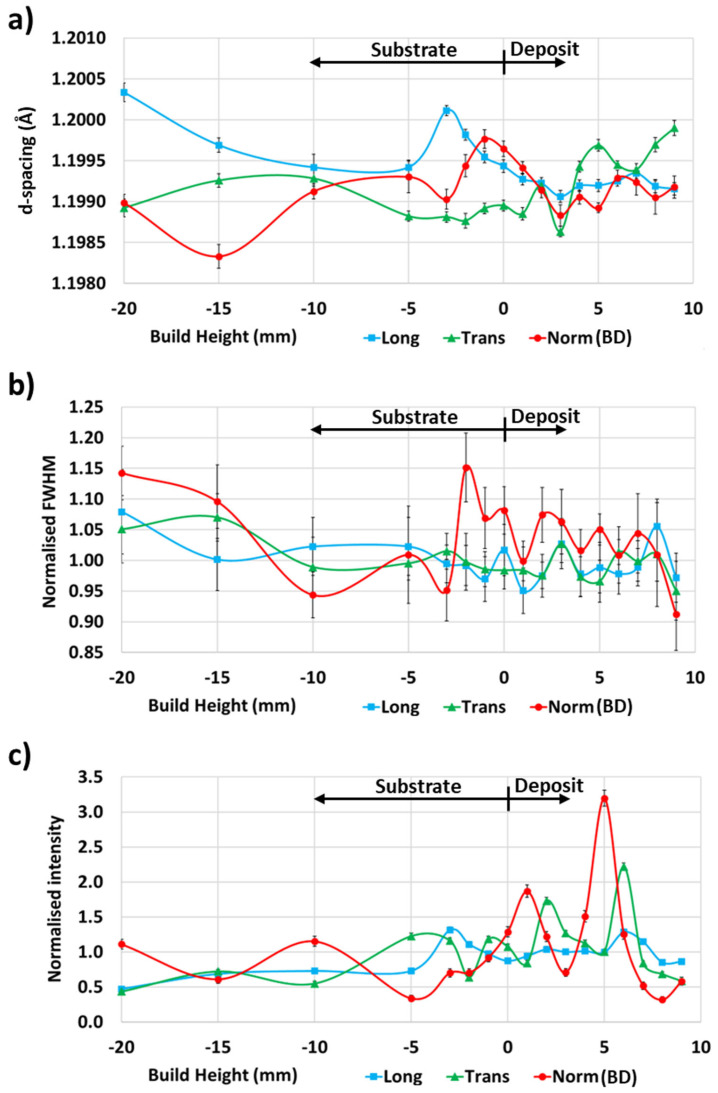
ND principal data for 10-pass deposit indicating (**a**) d∔spacing, (**b**) normalised FWHM, and (**c**) normalised intensity.

The measured lattice d-spacing for the 10-pass deposit in Figure 7a showed a clear positional dependence in the deposit and substrate in each principal direction. Furthermore, the d-spacing reached a local minimum at the 3 mm deposit height, reflecting the trend in d_0_-spacings observed in Figure 6. The same phenomenon was noted for the 4-pass and 7-pass deposits. While the d-spacing is an indicator of applied strain, in Al alloys, the d-spacing is also affected by the alloying element’s dissolution as well as precipitate formation as demonstrated by Lombardi et al. [54], necessitating the identification and use of strain-free d_0_-spacings for residual stress calculation.

Figure 7b shows the normalised 10-pass deposit neutron diffraction peak full-width half maxima (FWHM) for the longitudinal, transverse, and normal principal directions. The sharp increase in the normalised FWHM for the normal principal direction at the −2 mm build height indicates that the AFSD process caused a significant increase in plastic deformation and dislocation density at this location [55]. Since this region is in the substrate, it is assumed that it is most influenced by the first deposited layer. Furthermore, a relatively high normalised FWHM was seen for the normal direction from −2 mm to 8 mm build height of the 9.5 mm high deposit, indicating that the plastic deformation and dislocation density increase occurred in the preceding layers during subsequent deposition passes. ND was conducted along the deposit centreline, located directly under the compressive force (acting in normal principal direction) of the AFSD feed rod and at the centre of tool rotation, which is assumed to be the reason for the longitudinal and transverse directions experiencing no similar FWHM increase.

Meanwhile, Figure 7c shows normalised intensity spikes in the deposit not seen in the substrate. The intensity is related to the neutron beam path, grain size, texture, and other factors [56]. AFSD is known to form bands of highly-refined grains; however, they are not regularly distributed [46,57]. As such, the spikes in normalised intensity may be caused by the neutron beam interacting with highly-refined grain bands in the deposit, providing a large number of grains oriented correctly to capture Al(311) reflection. However, further research is required for confirmation.

**Figure 8:**
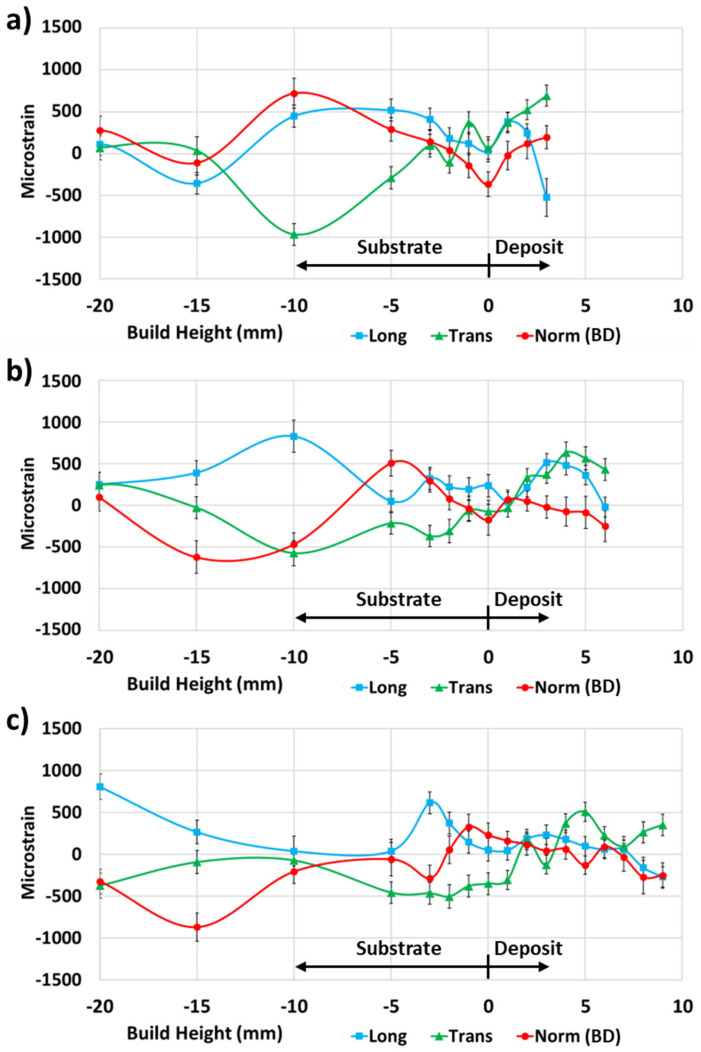
ND principal microstrain data for (**a**) 4-pass, (**b**) 7-pass, and (**c**) 10-pass deposits.

The microstrains for the 4-pass deposit (Figure 8a), 7-pass deposit (Figure 8b), and 10-pass deposit (Figure 8c) all showed relatively large difference in values for the longitudinal, transverse, and normal principal directions from 3 mm into the substrate to the bottom of the substrate. Meanwhile, 3 mm in the substrate for the four-pass deposit (Figure 8a), the strain values were relatively low and increased further to the top of the deposit. For the seven-pass deposit (Figure 8b), the strain values were approximately zero 1 mm into the deposit, increased 4 mm into the deposit, and decreased again to the top of the deposit. For the 10-pass deposit (Figure 8c), the strain values were approximately zero 2 mm into the deposit and remained relatively low to the top of the deposit.

**Figure 9:**
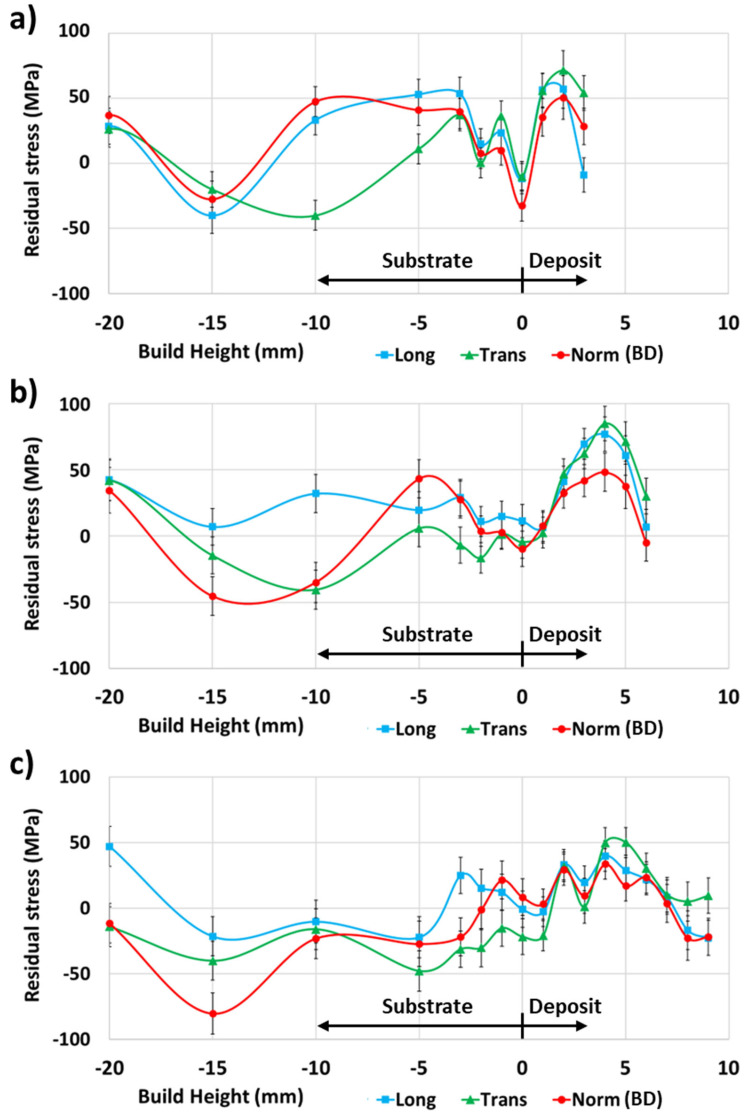
ND principal residual stress data for (**a**) 4-pass, (**b**) 7-pass, and (**c**) 10-pass deposits.

The neutron diffraction residual stress measurements of the 4-pass deposit (Figure 9a), 7-pass deposit (Figure 9b), and 10-pass deposit (Figure 9c) did not indicate a significantly higher residual stress magnitude in the deposit relative to the substrate. In all cases, the residual stress in the deposit was predominantly tensile, with the transverse principal direction exhibiting the highest residual stress magnitude of 72 MPa for the 4-pass deposit, 85 MPa for the 7-pass deposit, and 50 MPa for the 10-pass deposit. The interface between the deposit and substrate contained low residual stress relative to other measured areas.

**Figure 10:**
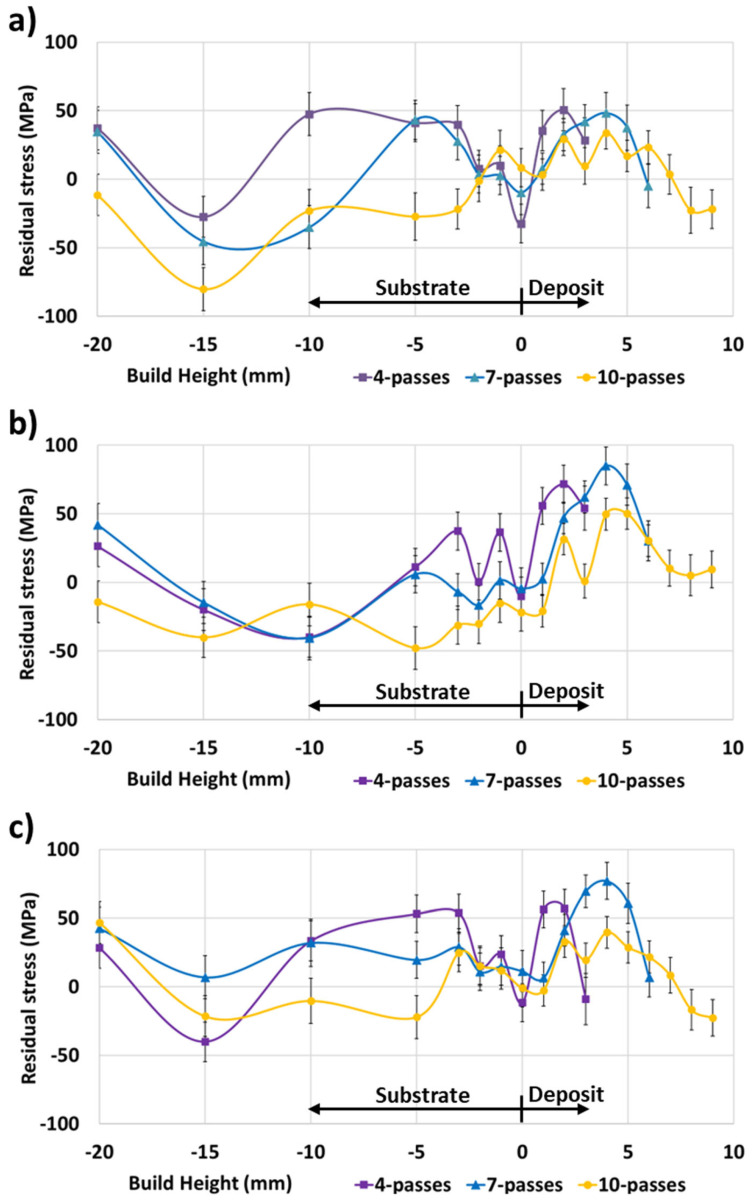
The 4-pass, 7-pass, and 10-pass deposit ND residual stress data for the (**a**) normal (build direction), (**b**) transverse, and (**c**) longitudinal principal directions.

Meanwhile, the substrate of the four-pass deposit (Figure 9a) exhibited predominantly tensile residual stress with a maximum magnitude of 54 MPa in the longitudinal principal direction. The substrates of the 7-pass (Figure 9b) and 10-pass (Figure 9c) deposits exhibited predominantly compressive residual stress, with a maximum magnitude of 45 MPa and 80 MPa, respectively, in the normal principal direction.

Comparing the normal (Figure 10a), transverse (Figure 10b), and longitudinal (Figure 10c) principal directions for the 4-pass, 7-pass, and 10-pass deposits, the substrate compressive residual stress appeared to balance the deposit tensile residual stress. Meanwhile, the interface between the deposit and substrate contained relatively low residual stress, with the residual stress increasing to the middle of the deposit before decreasing again at the top of the deposit.

In welded structures, the residual stresses are considered to directly impact the load-bearing capacity since loading stresses are superimposed over residual stresses [34]. Furthermore, tensile residual stresses increase the crack propagation rate, reducing the structure’s fatigue life [58,59]. From data in Figure 9a–c, no part of the structure is seen to contain significant residual stress relative to the reported room-temperature Al6061-T6 yield strength of 318 MPa [60]. However, the significant softening noted in Figure 4a–c indicates that a reduction in yield strength occurred. Qiao et al. [22] reported that deposits from an Al6061-T6 feedstock exhibited a yield strength range of 79.7–106 MPa, which was dependent on the deposition parameters. Meanwhile, for the same feedstock material, Tang et al. [23] reported a yield strength of 140 MPa for a tensile sample extracted from the final layer of the deposit and 113 MPa for a tensile sample extracted near the first layer of the deposit. The magnitude of the residual stresses in the 4-pass, 7-pass, and 10-pass deposits may, thus, be a significant fraction of the material’s yield strength in the as-manufactured condition.

Post-processing T6 heat treatment of AFSD-manufactured Al6061 has been shown to successfully restore the mechanical properties to those expected for the T6 condition [61]. For Al6063, the post-processing T6 heat treatment was reported to eliminate the hardness gradient similar to that noted in Figure 4a–c [21]. The solutionising step of the T6 heat treatment process exposes the material to a temperature greater than 500 °C [61], which softens the material and allows for plastic flow and residual stress relief. Meanwhile, during the quenching and ageing steps, a different residual stress profile is expected to form, which is dependent on the structure geometry [62].

## 4. Conclusions

This research provides an investigation of the microstructure, hardness, defects, and residual stress distribution as function of an increasing number of passes for an as-manufactured AFSD Al6061 stepped structure with 4-pass (3.5 mm height), 7-pass (6.5 mm height), and 10-pass (9.5 mm height) deposits.

The results are summarised as follows:

1. The microstructure of an AFSD deposit was characterised by refined grains and the presence of circular Fe and Si inclusions. While the deposit was well bonded to the substrate at the defect-free zone, comprising most of the deposit, the deposit and substrate interface presented no bonding at the flash zone. This is attributed to insufficient compressive force into the substrate in this area to result in effective mixing between the deposit and substrate materials. Furthermore, a large tunnel defect existed between the deposit and substrate at the flash zones, which can be machined off if those defects become critical.
2. A hardness gradient was observed for all deposits, which can be described as two hardness zones. Spanning 6 mm into the deposit at the deposit centreline from the top of the deposit, a zone of moderate hardness was noted, with the size of this zone remaining approximately constant for all deposit heights. Further into the deposit and into the substrate, a soft zone was noted, with the size of this zone becoming larger with increased deposit height. Furthermore, the deposit being offset to the side of the substrate resulted in relatively greater softening of the substrate in the offset direction.
3. The residual stresses in the deposits were predominantly low-magnitude tensile, while the residual stresses in the substrate were predominantly low-magnitude compressive. The hardness gradient indicates reduced material yield strength relative to T6 condition, and the deposit yield strength values reported in the literature allude to the deposit residual stress being a significant fraction of the yield strength of the deposit.

The residual stress characterisation of the Al6061 deposit indicated that the interface between the substrate and deposit did not contain significant residual stress.

These results can be vital for the development of finite element models to predict the residual stress distribution, microstructure evolution, and mechanical properties of AFSD deposits and can aid in process optimisation and in the design of new manufacturing strategies. A further enhancement of the understanding of the AFSD process and the expansion of its applications in various industries will lead to the development of high-performance, reliable, and cost-effective manufacturing solutions for single-phase alloys and composite-graded metal structures.

## Figures and Tables

**Table 1:** Two-dimensional area of defect-free, left-edge flash, and right-edge flash zones shown in Figure 2a–c calculated using ImageJ. Only the deposit area is measured.

Zone	4 Passes		7 Passes		10 Passes	
	Area (mm^2^)	% of Total Area	Area (mm^2^)	% of Total Area	Area (mm^2^)	% of Total Area
Defect free	105.40	82.0	231.29	86.3	368.41	83.5
Left-edge flash	12.87	10.0	17.80	6.7	31.17	7.1
Right-edge flash	10.24	8.0	18.79	7.0	41.76	9.4

## Data Availability

The data presented in this study are available on request from the corresponding author. The data are not publicly available due to privacy restrictions.
